# *Myrtus communis* Essential Oil; Anti-Parasitic Effects and Induction of the Innate Immune System in Mice with *Toxoplasma gondii* Infection

**DOI:** 10.3390/molecules26040819

**Published:** 2021-02-04

**Authors:** Raafat M. Shaapan, Hiba Riyadh Al-Abodi, Abdullah D. Alanazi, Sobhy Abdel-Shafy, Marzieh Rashidipour, Abdullah F. Shater, Hossein Mahmoudvand

**Affiliations:** 1 Department of Zoonosis, Veterinary Research Division, National Research Centre, El-Tahrir Street, Dokki, Giza 12622, Egypt; rmshaapan2005@yahoo.com; 2 Department of Environment, College of Science, University of Al-Qadisiyah, P.O. Box 88, Al-Diwaniyah 58001, Iraq; hiba.al-abodi@qu.edu.iq; 3 Department of Biological Science, Faculty of Science and Humanities, Shaqra University, Ad-Dawadimi 11911, Saudi Arabia; aalanazi@su.edu.sa; 4 Department of Parasitology and Animal Diseases, Veterinary Research Division, National Research Centre, Dokki, Giza 12622, Egypt; aasobhy@yahoo.com; 5 Nutritional Health Research Center, Lorestan University of Medical Sciences, Khorramabad 68149-93165, Iran; m_rashidi8001@yahoo.com; 6 Department of Medical Laboratory Technology, Faculty of Applied Medical Sciences, University of Tabuk, Tabuk 71491, Saudi Arabia; ashater@ut.edu.sa; 7 Razi Herbal Medicines Research Center, Lorestan University of Medical Sciences, Khorramabad 68149-93165, Iran

**Keywords:** chronic toxoplasmosis, herbal medicines, essential oils, *Myrtus communis*, *Toxoplasma gondii*

## Abstract

Background: *Myrtus communis* (*M. communis*) is a wild aromatic plant used for traditional herbal medicine that can be demonstrated in insecticidal, antioxidant, anti-inflammatory, and antimicrobial activity of its essential oils (MCEO). Aim: The present study aimed to evaluate the prophylactic effects of *M. communis* essential oil (MCEO) against chronic toxoplasmosis induced by the Tehran strain of *Toxoplasma gondii* in mice. Methods: Gas chromatography/mass spectrometry (GC/MS) analysis was performed to determine the chemical composition of MCEO. Mice were then orally administrated with MCEO at the doses of 100, 200, and 300 mg/kg/day and also atovaquone 100 mg/kg for 21 days. On the 15th day, the mice were infected with the intraperitoneal inoculation of 20–25 tissue cysts from the Tehran strain of *T. gondii*. The mean numbers of brain tissue cysts and the mRNA levels of IL-12 and IFN-γ in mice of each tested group were measured. Results: By GC/MS, the major constituents were α-pinene (24.7%), 1,8-cineole (19.6%), and linalool (12.6%), respectively. The results demonstrated that the mean number of *T. gondii* tissue cysts in experimental groups Ex1 (*p* < 0.05), Ex2 (*p* < 0.001) and Ex3 (*p* < 0.001) was meaningfully reduced in a dose-dependent manner compared with the control group (C2). The mean diameter of tissue cyst was significantly reduced in mice of the experimental groups Ex2 (*p* < 0.01) and Ex3 (*p* < 0.001). The results demonstrated that although the mRNA levels of IFN-γ and IL-12 were elevated in all mice of experimental groups, a significant increase (*p* < 0.001) was observed in tested groups of Ex2 and Ex3 when compared with control groups. Conclusion: The findings of the present study demonstrated the potent prophylactic effects of MCEO especially in the doses 200 and 300 mg/kg in mice infected with *T. gondii*. Although the exceptional anti-*Toxoplasma* effects of MCEO and other possessions, such as improved innate immunity and low toxicity are positive topics, there is, however, a need for more proof from investigations in this field.

## 1. Introduction

Toxoplasmosis is one of the most prevalent zoonotic parasitic diseases caused by the intracellular parasite *Toxoplasma gondii* (*T. gondii*). This parasite affects more than 30% of the world’s population and a wide range of warm-blooded animals [1]. Human as the intermediate host is infected through main routes, including: (i) the ingestion of undercooked or uncooked meat infected with tissue cysts of *T. gondii*, (ii) consumption of water and food contaminated with sporulated oocysts excreted in feces of the cat as definitive host, and (iii) congenital infection, when the mother becomes infected during pregnancy by one of two previous methods [2,3]. Considering clinical manifestations of toxoplasmosis, the disease does not cause any specific symptoms in healthy and immunocompetent people; but, a severe and even a deadly form can be observed in immunocompromised individuals (such as patients with human immunodeficiency virus (HIV)/acquired immune deficiency syndrome (AIDS), and patients with organ transplantation, etc.) and congenitally infected fetuses [4].

At present, chemotherapy with the combination of pyrimethamine and sulfadiazine followed by azithromycin, clindamycin, atovaquone, etc., is considered as the preferred treatment for toxoplasmosis; however, studies in recent years have demonstrated that these drugs are associated with some side effects such as osteoporosis, and teratogenic effects mostly in immune-compromised patients [5,6]. Atovaquone, a hydroxynaphthoquinone derivative, has potent activity against tissue cysts through the blocking respiratory chain of the *Toxoplasma*; therefore, it is broadly used for in vivo activity against *T. gondii* during both infection stages [7].

Since there is currently no effective vaccine to prevent toxoplasmosis in human and animals, prophylaxis can therefore be considered the best way to prevent the toxoplasmosis, especially in immunocompromised individuals with a CD4 count below 100 cells/μL as well as in pregnant women who were not previously determined to be seronegative for *Toxoplasma* Immunoglobulin G (IgG) [8,9].

From ancient times, medicinal herbs and their derivatives have been broadly used for health promotion and therapy for chronic, as opposed to life-threatening, diseases [10,11]. Herbal medicines have also been successfully used in the treatment of a wide range of bacterial, viral, fungal, as well as parasitic infections [12,13,14,15,16]. *Myrtus communis* L. (*M. communis*), which also called myrtle (*Myrtaceae* family) is a medicinal herb that has been broadly applied for folk medicine around the world [17]. Since the old civilizations, myrtle has long been applied in traditional medicine as a reliever of stomach aches, wound healing, antihemorrhoid, etc. [18]. Recently, modern medicine demonstrated that various parts of this plant such as leaves, fruits, roots, berries, and its branches have different pharmacological possessions including anti-inflammatory, analgesic, antioxidant, anticancer, anti-diabetic, anti-mutagenic, neuro-protective, etc. [19]. Numerous studies have also reported antimicrobial effects of *M. communis* against a wide range of pathogenic strains of bacteria (*Staphylococcus aureus, Listeria monocytogenes, Pseudomonas aeruginosa, Escherichia coli, Klebsiella pneumonia*, etc.), viruses (*Herpes simplex*), fungi (*Candida* spp., etc), and parasites [19,20,21,22]. From a long time ago, essential oil and its constituents are considered as a promising therapeutic agent, due to their qualified safety, and broad biological and pharmacological activities [23]. Reviews have shown that essential oil of *M. communis* contains a large amount of are terpenes, terpenoids, and phenylpropanoids [17].

Given the various pharmacological effects of *M. communis*, the present study aimed to evaluate the prophylactic effects of *M. communis* essential oil against chronic toxoplasmosis induced by the Tehran strain of *T. gondii* in mice.

## 2. Results

### 2.1. GC/MS Analysis

The yield of essential oil was 0.41 % (*v*/*w*). Density of essential oil at 25 °C was 0.831 g/mL and refractive index was 1.391 at 25 °C. Based on the obtained results in GC/MS, twenty-five compounds were identified, indicating 93.01% of the total essential oil (Table S1, Supplementary Materials). The major constituents were *α*-pinene (24.7%), 1,8-cineole (19.6%), and linalool (12.6%), respectively.

### 2.2. Parasitological Study

#### 2.2.1. The Mean Number of *T. gondii* Tissue Cysts

**Figure 1:**
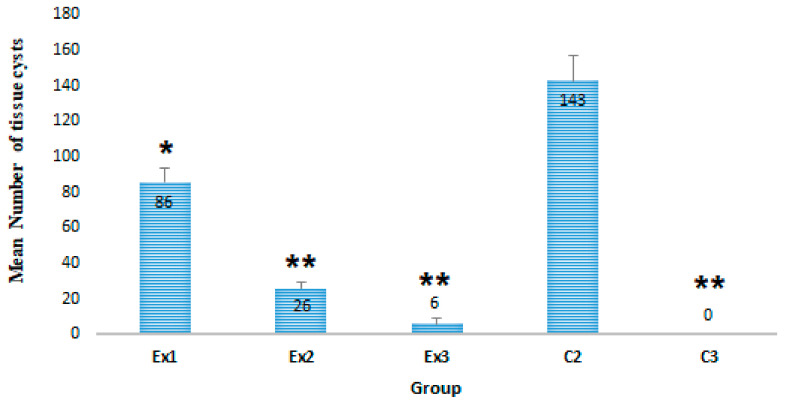
The mean numbers of brain tissue cysts in mice of tested group. The results demonstrated that the mean number of *T. gondii* tissue cysts in experimental groups Ex1 (*p* < 0.05), Ex2 (*p* < 0.001), and Ex3 (*p* < 0.001) was meaningfully reduced as a dose-dependent manner compared with control group (C2). * *p* < 0.05, ** *p* < 0.001.

Figure 1 shows the frequency of the brain tissue cysts in tested mice of each group. Based on the obtained findings, oral administration of MCEO for 3 weeks significantly decreased the mean number of *T. gondii* tissue cysts mice of the tested groups of Ex1 (*p* < 0.05), Ex2 (*p* < 0.001), and Ex3 (*p* < 0.001), in comparison with the control group (C2).

#### 2.2.2. The Mean Diameter of *T. gondii* Tissue Cysts

**Figure 2:**
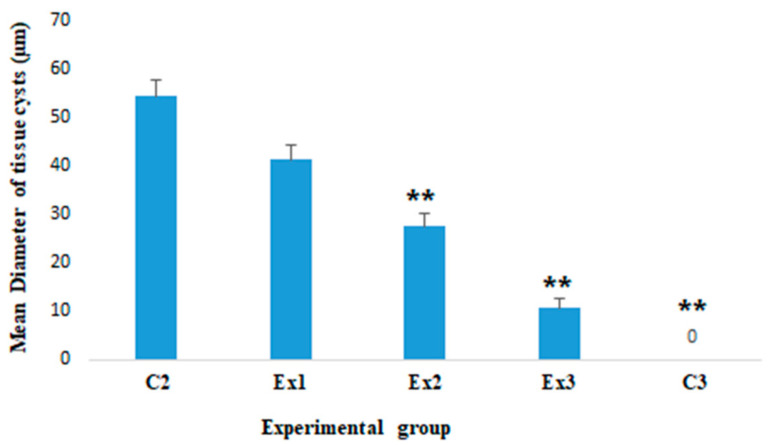
The mean diameter of brain tissue cysts in mice of tested group. The mean diameter of tissue cysts in experimental group C2 was 57.4 ± 3.35 µm, although this value was 43.5 ± 2.96 µm in mice of experimental group Ex1 (100 mg/kg); however, the mean diameter of tissue cyst was significantly reduced in mice of experimental groups Ex2 (200 mg/kg) and Ex3 (300 mg/kg). ** *p* < 0.001.

By the mean diameter of *T. gondii* tissue cysts, the results exhibited that the mean diameter of tissue cysts in experimental group C2 was 57.4 ± 3.35 µm, although this value was 43.5 ± 2.96 µm in mice of experimental group Ex1; however, the mean diameter of tissue cyst was significantly reduced in mice of experimental groups Ex2 (*p* < 0.01) and Ex3 (*p* < 0.001) (Figure 2).

### 2.3. Cytokine Expression by Real-Time PCR

**Figure 3:**
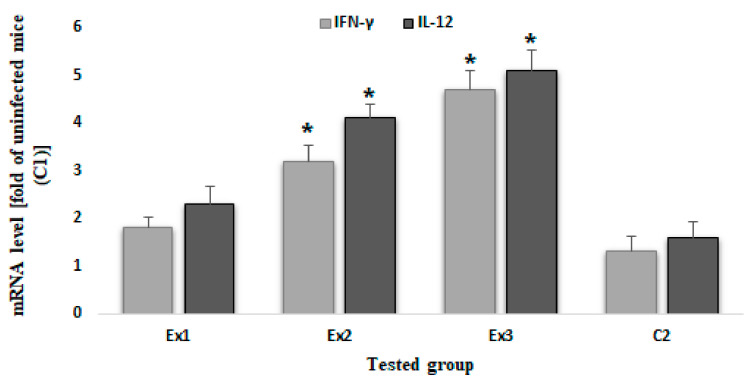
The expression level of genes producing IFN-γ and IL12 cytokines in mice treated with MCEO. The results demonstrated that although the mRNA levels of IFN-γ and IL-12 were elevated in all mice of experimental groups, a significant increase (*p* < 0.001) was observed in tested groups of Ex2 (200 mg/kg) and Ex3 (300 mg/kg), when compared with control groups. * *p* < 0.05.

Figure 3 shows the mRNA levels of IFN-γ and IL-12, in mice of all tested groups. The results demonstrated that although the mRNA levels of IFN-γ and IL-12 were elevated in all mice of experimental groups, a significant increase (*p* < 0.001) was observed in tested groups of Ex2 and Ex3, when compared with control groups.

## 3. Discussion

At present, the combination of sulfonamide and pyrimethamine is considered the gold-standard therapy to treat toxoplasmosis [24]. According to the reports, these are associated with adverse side effects and responses such as teratogenic effects, hematological disorders, myelosuppression, and gastrointestinal effects, etc [5,6]; hence, the discovery of novel effective agents especially from natural sources with low toxicities is an absolute need.

The World Health Organization (WHO) reported that more than two-third of the world’s population trust in folk medicine for their early therapeutic purposes [25]. Reviews demonstrated that herbs used for therapeutic purposes in traditional medicine contain a variety of compounds that have different biological and therapeutic activities especially in the treatment of microbial infections [26]. Here, we aimed to assess the prophylactic effects of *M. communis* essential oil against chronic toxoplasmosis induced by the Tehran strain of *T. gondii* in mice.

The obtained parasitological results demonstrated the exceptional anti-*Toxoplasma* effects of MCEO, so that the mean number of *T. gondii* tissue cysts in experimental groups of Ex1 (*p* < 0.05), Ex2 (*p* < 0.001), and Ex3 (*p* < 0.001) was meaningfully reduced in a dose-dependent manner compared with the control group (C2). The results also exhibited that the mean diameter of tissue cyst was significantly reduced in mice of experimental groups Ex2 (*p* < 0.01) and Ex3 (*p* < 0.001).

Considering the antiparasitic activity of *M. communis,* Mahmoudvand et al. (2015) have demonstrated that the essential oil and methanolic extract of *M. communis* significantly suppressed the growth percentage of promastigote and amastigote forms of *Leishmania tropica* with the IC_50_ values ranging from 8.4 to 40.8 μg/mL [20]. In the other study conducted by Azadbakht et al. (2004), the results showed that essential oil and methanolic extract of *M. communis* at the concentrations of 0.0001 to 0.1 considerably reduced the growth rate of *Trichomonas vaginalis* trophozoites on in vitro experiments [27]. The results of another study showed that MCEO at the dose of 12.5 to 100 μg/mL significantly reduced the viability of protoscoleces of *Echinococcus granulosus* in vitro [28]. The anti-plasmodial effects of methanolic extract of *M. communis* were demonstrated against chloroquine-resistant (K1) and chloroquine-sensitive (3D7) strains of *Plasmodium falciparum* with the IC_50_ values of 35.44 and 0.87 µg/mL, respectively [29]; the authors also concluded that *M. communis* methanolic extract considerably reduced the parasitemia in mice infected with *Plasmodium berghei* after 4 days of treatment.

Although the chemical composition of MCEO was studied in different studies around the world [21], it has been proven that the chemical composition of essential oils is somewhat variable depending on some factors such as the plant collection place, part of used, the time of harvest, and the method of extraction [30]. Based on the previous studies, terpenoid compounds such as 1,8-cineole, α-pinene, limonene, linalool, α-terpinolene, etc., are considered as the main components found in *M. communis* essential oil [31]. The results of our study in agreement with previous studies show that the major constituents of MCEO were *α*-pinene (24.7%), 1,8-cineole (19.6%), and linalool (12.6%), respectively [32]. Reviews have demonstrated that the antiviral, antibacterial, antifungal, and anti-parasitic activities of terpenes, terpenoides, and their derivatives against a wide range of pathogenic strains [19]. It this indicated that these phytoconstituents could be responsible for their antimicrobial activity while their precise mechanism of action is not clearly understood. Previous studies have demonstrated that these compounds showed antimicrobial effects through the disruption of cell membrane, inhibition of oxygen consumption, inhibition of virulence factors, etc. [26].

Since one of the most important mechanisms of control of toxoplasmosis is the immune system, mainly cellular immunity, we evaluated the mRNA levels of some innate immunity mediators such as IFN-γ and IL12 by quantitative real time PCR [33]. The results demonstrated that although the mRNA levels of IFN-γ and IL-12 were elevated in all mice of experimental groups, a significant increase was observed in mice treated by MCEO at the doses of Ex2 and Ex3 of MCEO when compared with control groups. With respect to the immunomodulatory effects, it has been proven that cytokine IL-12 controls nitric oxide synthesis via IFN-γ. In *T. gondii* infection, the production of nitric oxide is controlled by the partial inhibition of the synthesis of nitric oxide synthetase. Theoretically, modulation of cytokine is extremely important, because nitric oxide is considered as a part of the initial effectors in the immune system response against toxoplasmosis [34]. Our findings suggest that the decrease in parasite load in the infected mice treated with MCEO can be associated to the strengthening of the immune system, principally the innate immune system, of the tested mice that result in the control of *T. gondii* infection. Considering the toxicity of MCEO, in the study conducted by Mahmoudvand et al. (2016), the results showed that there was no significant toxicity in the clinical chemistry and hematological parameters after 14 days of oral administration of MCEO at the doses 0.05, 0.1, 0.2, and 0.4 mL/kg in tested mice, indicating that MCEO at the tested doses of the present study has no toxicity in BALB/c mice [28].

Although the present investigation showed that the exceptional anti-*Toxoplasma* effects of MCEO has been proven, several important points must be considered in the use of plant products, including the use of a standard method for preparation of essential oil, the proper selection of concentrations or dilutions, the finding of the most active fraction or extracts, selection of the type of study to better investigate the mechanism of action, the study of the pharmacokinetic profile of the plant products, etc. [35].

## 4. Materials and methods

### 4.1. Collecting the Plant Materials

In this investigation, the leaves of plant were prepared from mountain areas of Kerman province in September 2016. After identifying the plant by a botanist, a voucher sample of the plant materials was placed at the Herbarium of Department of Pharmacognosy of School of Pharmacy, (Kerman, Iran) (KF1356).

### 4.2. Isolation of the Essential Oil

About 500 g of air-dried leaves were used to hydro-distillation for 3 h by means of an all-glass Clevenger-type device. The obtained essential was dried over anhydrous sodium sulfate and kept in darkness at 4 °C in air-tight glass vials closed under nitrogen gas until testing [36].

### 4.3. Gas Chromatography/Mass Spectrometry (GC/MS) Analysis of Essential Oil

A Hewlett-Packard 6890 (Hewlett-Packard, Palo Alto, CA, USA) apparatus was applied to perform the GC analysis equipped with a HP-5MS column (30 m × 0.25 mm, film thickness 0.25 mm). Other device specifications and processes such as column temperature, injector and interface temperatures, flow rate of helium, etc., were previously described in the study conducted by Mahmoudvand et al. [28]. To determine the chemical composition of the essential oil we evaluated the relative retention time and mass spectra of each detected compound compared with the standards Wiley 2001 library data or literature ones [31].

### 4.4. Experimental Design and Infection

#### 4.4.1. Animals

A total of 48 male BALB/c mice (6–8 weeks old) weighing from 20 to 25 g were used in this study. Mice were housed in a colony room with a 12:12 h light/ dark cycle at 21 ± 2 °C and maintained with free access to water and feeding ad libitum. Mice were handled based on the standard protocols for the use of laboratory animals [37]. 

#### 4.4.2. Parasite

In this survey, the Tehran strain of *T. gondii* (type II) was kindly prepared from the strain kindly obtained from Prof. Hossein Keshavarz and Prof. Saeedeh Shoajee (Tehran University of Medical Sciences, Tehran, Iran). The parasites were passaged via intraperitoneal injection of 15–20 tissue cysts every 90 days into new BALB/c mice.

#### 4.4.3. Animal Model of Chronic Toxoplasmosis

The chronic model of toxoplasmosis in mice was induced based on the method described previously [37]. To do this, 0.5 mL of brain homogenized suspension (obtained from infected mice) having as a minimum 25–30 tissue cysts with antibiotics of penicillin and streptomycin were injected intraperitoneal to mice of each studied group.

#### 4.4.4. Design

**Figure 4:**
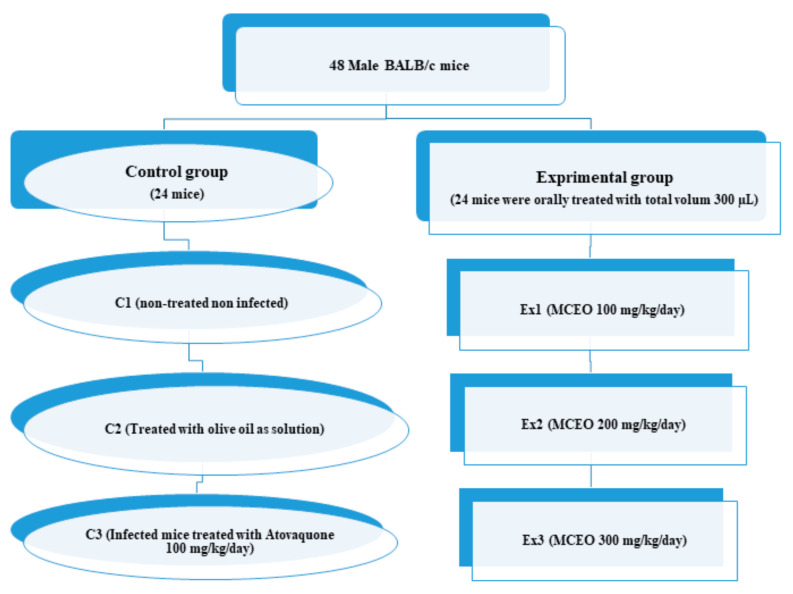
The flowchart of study design of the present study.

Figure 4 shows the experimental design of the present study. Male BALB/c mice were divided into two main groups (control (C) and experimental group (Ex)) with six sub-groups including C1 (non-treated non infected), C2 (treated with olive oil as solvent), C3 (Infected mice treated with Atovaquone 100 mg/kg/day), Ex1 (MCEO 100 mg/kg/day), Ex2 (MCEO 200 mg/kg/day), and Ex3 (MCEO 300 mg/kg/day). After 3 weeks of treatment, the mice in all groups, except the C_1_ group, were infected with the Tehran strain of *T. gondii.* It should also be mentioned that the selection of doses of MCEO was based on the previous study conducted by the present authors which revealed that MCEO in these doses has no toxicity in mice [28].

### 4.5. Serological Tests

To confirm the development of toxoplasmosis in mice, serum samples of mice from each tested group was collected for evaluation of anti-*T. gondii* IgG antibody by a modified agglutination test (MAT) kit (Toxo screen DA, Biomérieux, Lyon, France), the formalized killed whole tachyzoites of *T. gondii* was prepared and procedures were carried out according to the method described by Shaapan et al. [38]. The agglutination titer of ≤1/20 was positive and end-titrated by 2-fold dilutions.

### 4.6. Sample Collection

To collect the brain samples, mice in each group were deeply anesthetized by means of intraperitoneal administration of ketamine (150 mg/kg) and xylazine (10 mg/kg). In the next step followed by decapitation, total brain tissues from each mouse were aseptically collected. To evaluate the parasitological changes, we applied the right hemisphere; while another hemisphere was maintained in −80 °C to determine the molecular examinations.

### 4.7. Anti-Parasitic Activity

To assess the effects of MCEO on *T. gondii* infection, the right hemisphere of brain from each mouse was used to prepare the unstained-smears; in the next step, the diameter and the numbers of tissue cysts were calculated at two magnifications of 100× and 400× by means of light microscopy [39].

### 4.8. Induction of Innate Immune System

The mRNA levels of IFN-γ, and IL12 which are considered as key factors related to toxoplasmosis control mechanisms were measured in all tested mice using quantitative real time PCR. The total brain RNA was extracted by means of the RNA-easy kits (Qiagen, Hilden, Germany); whereas all isolated RNAs were reverse-transcribed according to the manufacture’s protocols. Consequently, the collected complementary DNA (cDNA) was applied to conventional PCR amplification or real-time PCR. To perform the Real-time PCR we used the iQ5 real-time PCR detection system (Bio-Rad, Hercules, CA). All amplification products were determined by SYBR green [40]. The reaction conditions of real-time PCR were included initial denaturation at 95 °C for 10 min, 40 amplification cycles [denaturation at 95 °C for 10 s, annealing at 56 °C for 30 s, and elongation at 72 °C for 30 s], followed by one cycle at 72 °C for 5 min. The iQ^TM^5 optical system software (Bio-Rad) was used for data analysis. Here, β-actin which is well-known as a housekeeping gene was considered as a normalization control. Oligonucleotide primers used for real-time RT-PCR analysis are shown in Table 1.

### 4.9. Statistical Analysis

Data analysis was carried out using SPSS statistical package version 17.0 (SPSS Inc., Chicago, IL, USA). One-way ANOVA with Turkey’s potshot test was used to assess differences between experimental groups. In addition, *p* < 0.05 was considered statistically significant.

## 5. Conclusions

The findings of the present study demonstrated the exceptional anti-*Toxoplasma* effects of MCEO in infected mice with *T. gondii*. Thus, oral administration of MCEO in the doses of 200 and 300 mg/kg for 21 days was able to prevent severe symptoms of the toxoplasmosis in the mouse model. However, the exceptional anti-*Toxoplasma* effects of MCEO and other effects, such as improved innate immunity and low toxicity, are positive topics that need more proof from investigations in this field.

## Figures and Tables

**Table 1:** Sequences of primers used for real-time PCR.

Amplicon	Primers	Sequence (5′–3′)
IL12	F	ACGACATTCGTCAACTGCAA
	R	TAAATTGGCACCCTGTAGGC
IFN-γ	F	GATCGTGTCGTCACCAGAAAGG
	R	TGCCTGGTAACGAGTTGTCC

## Data Availability

All data generated or analyzed during this study are included in this published article.

## Supplementary Materials

The following are available online, Table S1: Essential oil composition of *M. communis* identified by GC/MS.

## Sample Availability

Samples of the compounds are available from the authors.
