# Supplementary material for: Myrtus communis Essential Oil; Anti-Parasitic Effects and Induction of the Innate Immune System in Mice with Toxoplasma gondii Infection
Source: Molecules. 2021 Feb 4;26(4):819. doi: 10.3390/molecules26040819 (PMC7915315; doi:10.3390/molecules26040819)
Supplement: Supplementary file 1 [file molecules-26-00819-s001.pdf]

Table S1. Essential oil composition of *M. communis* identified by GC/MS

No.	Compound	Percentage
1.	α-Thujene	0.88
2.	Camphene	0.58
3.	δ-3-Carene	0.73
4.	α-Pinene	24.7
5.	β –Pinene	1.28
6.	β –Myrcene	0.61
7.	α –Terpinene	0.23
8.	1,8- Cineole	19.6
9.	Methyl eugenol	1.3
10.	Linalool	12.6
11.	α-Terpinyl acetate	3.8
12.	Myrtenyl acetate	8.3
13.	α –Phellandrene	0.1
14.	β –Ocimene	0.11
15.	2,6-Octadien	0.41
16.	α –Phellandrene	0.1
17.	γ-Terpinene	0.5
18.	α-Terpinolene	0.51
19.	4-Terpineol	0.6
20.	α-Terpineol	6.1
21.	Linalyl Acetate	5.9
22.	Caryophyllene oxide	1.4
23.	α-Humulene	1.2
24.	Neryl acetate	0.14
25.	trans-Caryophyllene	1.33
	**Total**	**93.01**
